# Community-Engaged Older Adult-Led Policy, Systems, and Environment Interventions in the MS High Obesity Program

**DOI:** 10.1093/geroni/igab046.1620

**Published:** 2021-12-17

**Authors:** Masey Smith, Katie Halfacre, Megan Holmes, David Buys

**Affiliations:** 1 Mississippi State University, Starkville, Mississippi, United States; 2 Mississippi State University, MS State, Mississippi, United States; 3 Mississippi State University, STARKVILLE, Mississippi, United States

## Abstract

Older adults in rural areas are at unique risk for poor outcomes due to social isolation and limited access to resources. The Mississippi High Obesity Program (HOP) aims to enhance access to social connections and resources like community gardens, food pantries, and physical activity as part of its broader objective to prevent and reduce obesity. Through policy, systems and environment strategies, development of Memoranda of Understanding (MOUs) between aforementioned entities, and community based participatory research approaches, Mississippi HOP efforts enhance food systems improvement efforts; grow multi-sectoral collaboration; and evaluate the effectiveness of new policies, and specifically MOUs, in reaching these goals. Older adults represent more than 40% (n=27) of all coalition members and stakeholder leaders (n=61); they are essential for the success of these initiatives. This presentation will highlight the work done during the COVID-19 pandemic and the role of and benefits to older adults, especially ones in rural communities.

